# Phase I/II study of Resiquimod as an immunologic adjuvant for NY-ESO-1 protein vaccination in patients with melanoma

**DOI:** 10.1186/2051-1426-1-S1-P272

**Published:** 2013-11-07

**Authors:** Rachel L Sabado, Anna Pavlick, Sacha Gnjatic, Crystal Cruz, Isabelita Vengco, Farah Hasan, Farbod Darvishian, Luis Chiriboga, Rose Marie Holman, Juliet Escalon, Caroline Muren, Crystal Escano, Ethel Yepes, Dunbar Sharpe, Sylvia Adams, Patrick Ott, Achim Jungbluth, Linda Pan, Ralph Venhaus, Nina Bhardwaj

**Affiliations:** 1 New York University School of Medicine, New York, NY, USA; 2 Mount Sinai Icahn School of Medicine, New York, NY, USA; 3 Ludwig Institute for Cancer Research, New York, NY, USA

## Purpose

The TLR 7/8 agonist, Resiquimod has been shown to induce local activation of immune cells, production of cytokines, and antigen-presentation by dendritic cells, features desirable for cancer vaccine adjuvants. In this study, we evaluated the safety and immunogenicity of vaccination with NY-ESO-1 protein emulsified in Montanide ISA-51 VG when given with or without Resiquimod in surgically resected stage IIB-IV melanoma patients.

## Experimental design

This is a two-part study design. Part I represents an open-label dose-escalation with Resiquimod using 2 cohorts treated with 100μg NY-ESO-1 protein emulsified in 1.25mL Montanide (day1) followed by topical application of 1000mg of the 0.2% Resiquimod gel on days 1 and 3 for cohort-1 (N=3) or days 1, 3, and 5 for cohort-2 (N=3). The cycles were repeated every 3 weeks, total of 4 cycles. For part II of the study, patients were blindly randomized to receive 100μg NY-ESO-1 protein emulsified in 1.25mL Montanide (day1) followed by topical application of placebo gel (Arm-A; N=8) or 1000mg of 0.2% Resiquimod gel (Arm-B; N=12) using the dosing regimen established in Part I. Blood samples were collected at baseline, one week after each cycle of vaccination, and at follow-up visit for the assessment of NY-ESO-1-specific humoral and cellular immune responses.

## Results

The vaccines were generally well-tolerated, with no grade 4 adverse events or study-related deaths. Most study participants experienced mild adverse reactions reported as Grade 1 or 2 per CTCAE criteria v. 4. One patient experienced a grade 3 syncopal episode that was unrelated to the study drugs and another patient had a grade 3 injection site necrosis that was possibly related to the study drugs. NY-ESO-1 specific antibody responses were induced in both study arms although higher mean antibody titers were observed in Arm B. NY-ESO-1 specific CD4+ T cell responses were induced in patients in both study arms. However, significant NY-ESO-1 CD8+ T cell responses were detected only in Arm B.

## Conclusions

The current study shows that Resiquimod is safe and contributes to the induction of immune responses in patients.

